# Is increased mutation driving genetic diversity in dogs within the Chornobyl exclusion zone?

**DOI:** 10.1371/journal.pone.0315244

**Published:** 2024-12-27

**Authors:** Megan N. Dillon, Allison N. Dickey, Reade B. Roberts, Jennifer A. Betz, Timothy A. Mousseau, Norman J. Kleiman, Matthew Breen

**Affiliations:** 1 Department of Molecular Biomedical Sciences, College of Veterinary Medicine, North Carolina State University, Raleigh, NC, United States of America; 2 Bioinformatics Research Center, North Carolina State University, Raleigh, NC, United States of America; 3 Department of Biological Sciences, North Carolina State University, Raleigh, NC, United States of America; 4 Visiting Veterinarians International, Damascus, OR, United States of America; 5 Department of Biological Sciences, University of South Carolina, Columbia, SC, United States of America; 6 Department of Environmental Health Sciences, Mailman School of Public Health, Columbia University, New York, NY, United States of America; 7 Comparative Medicine Institute, North Carolina State University, Raleigh, NC, United States of America; 8 Center for Human Health and the Environment, North Carolina State University, Raleigh, NC, United States of America; 9 Cancer Genetics, UNC Lineberger Comprehensive Cancer Center, University of North Carolina, Chapel Hill, NC, United States of America; 10 Duke Cancer Institute, Duke University, Durham, NC, United States of America; Institute of Physiology and Pharmacology, University of Agriculture Faisalabad, PAKISTAN

## Abstract

Environmental contamination can have lasting impacts on surrounding communities, though the long-term impacts can be difficult to ascertain. The disaster at the Chornobyl Nuclear Power Plant in 1986 and subsequent remediation efforts resulted in contamination of the local environment with radioactive material, heavy metals, and additional environmental toxicants. Many of these are mutagenic in nature, and the full effect of these exposures on local flora and fauna has yet to be understood. Several hundred free-roaming dogs occupy the contaminated area surrounding the Chornobyl Nuclear Power Plant, and previous studies have highlighted a striking level of genetic differentiation between two geographically close populations of these dogs. With this work, we investigate mutation as a possible driver of this genetic differentiation. First, we consider large-scale mutation by assessing the karyotypic architecture of these dogs. We then search for evidence of mutation through short tandem repeat/microsatellite diversity analyses and by calculating the proportion of recently derived alleles in individuals in both populations. Through these analyses, we do not find evidence of differential mutation accumulation for these populations. Thus, we find no evidence that an increased mutation rate is driving the genetic differentiation between these two Chornobyl populations. The dog populations at Chornobyl present a unique opportunity for studying the genetic effects of the long-term exposures they have encountered, and this study expands and builds on previous work done in the area.

## Introduction

Environmental contamination is a growing issue globally, as it is estimated that over 5,000 mass produced chemicals have been extensively dispersed into the environment and more than 22 million hectares of land are estimated to be affected by soil pollution [1, 2]. In the United States alone, the Environmental Protection Agency notes that approximately 73 million people live within three miles of a Superfund site, defined as a polluted location that requires long-term cleanup [3]. This means that 22% of the U.S. population may be exposed to various environmental toxicants and harmful chemicals. Exposure extends beyond the human populations, as countless domestic, food animal, and wildlife species also inhabit these polluted environments [4]. Toxicants found in these environments, including mercury, lead, and arsenic, can have detrimental health and genotoxic effects on both humans and animals [5, 6]. For example, at the Superfund site within Crab Orchard National Wildlife Refuge, researchers found a decrease in survival of European Starling chicks exposed to polychlorinated biphenyls (PCBs) compared to unexposed reference populations [7]. A growing area of research centers around understanding the long term and multigenerational effects of these contaminants on wildlife populations. In places of widespread radioactive contamination, such as Fukushima in Japan and Chornobyl in Ukraine, some previous studies linked DNA damage and reproductive effects in various kinds of fauna and flora to radiation exposure, including persistent genetic damage noted in insect species and sperm abnormalities in mice and barn swallows (reviewed in [8, 9]). Other studies have raised questions about the true extent of causal relationships between radiation exposure after these accidents and diminished reproductive potential or hereditary mutational events [10, 11].

Many environmental toxicants can also introduce mutation or cause damage to the DNA. Ionizing radiation induces mutations via improper repair of radiation induced DNA double-stranded breaks or base damage [12, 13]. Similarly, heavy or toxic metals as well as other environmental toxicants, cause DNA mutation via direct DNA-damaging mechanisms, or by indirect means such as increasing intracellular oxidative stress or interfering with DNA repair [5]. In the United States, for example, EPA-designated Superfund sites contain a wide variety of toxicants capable of inducing mutation and affecting both human and animal health. A critical question surrounding these contaminated sites is their potential effects on surrounding communities; what is the likelihood that prolonged exposure to low levels of anthropogenic health hazards will alter the mutation rate of the inhabitants or their offspring?

The area surrounding the Chornobyl Nuclear Power Plant complex in northeastern Ukraine is an extreme example of a contaminated environment. The 1986 nuclear disaster resulted in the world’s largest release of radioactive nuclides, including Iodine-131, Cesium-137 and Strontium-90 [14, 15]. However, the subsequent contamination was not limited solely to radioisotopes. Remediation efforts and the abandonment of multiple military and industrial complexes in the vicinity of the power plant resulted in significantly elevated levels of lead, arsenic, organics, pesticides, asbestos, and many other hazardous compounds in the surrounding areas [16–19]. Many of these toxicants increase mutagenic and/or carcinogenic risk to exposed plant and animal life [13, 20]. Shortly after the accident, the former Soviet authorities designated a Chornobyl Exclusion Zone (CEZ) of ~2,600 km^2^, extending to roughly a 30 km radius around the nuclear power plant that contained the most contaminated regions. The human population in the towns, villages, and cities in the CEZ were evacuated 24–36 hours after the disaster. While efforts were also made to “liquidate” both wildlife and domestic animals left behind within the CEZ, some survived and reproduced. These populations experienced multigenerational exposures to mutagenic hazards, including ionizing radiation, heavy metals, and organics [21–23]. Despite population growth noted in some of these local species, the full extent of the longitudinal, mutagenic impact of such exposures is poorly understood.

Considerable debate surrounds whether low-level, multi-generational toxic exposures in Chornobyl induced a higher DNA mutation rate. This is particularly relevant for the several hundred free-breeding dogs living within the CEZ, which are hypothesized to be the descendants of the original, domestic pets of the residents in the region [24]. Dogs, and other companion animals, may serve as effective sentinels for human health effects arising from toxic environmental exposures [25, 26]. Thus, Chornobyl dogs present a unique and valuable resource as models for human mutation studies. Previously, we found significant genetic differentiation between a population of dogs residing in and around the Chornobyl Nuclear Power Plant, where the levels of environmental contamination are higher, and a population only 16 km away in Chornobyl City [27, 28]. However, we detected similar breed compositions and individual inbreeding levels in these two dog populations, suggesting that these two factors are unlikely to be strong contributors to the genetic differences identified [27]. Additionally, this level of genetic differentiation was not found between other Eurasian free-breeding dog populations separated by more geographic distance [27]. These observations, collectively, suggest that genetic variation in our study populations cannot be attributed solely to geographic isolation, inbreeding, or breed discrepancies. In addition, there are no notable visual differences between these two populations. Both of these populations have occupied the CEZ for over 36 years, albeit at different distances from the damaged reactor, the epicenter of the contamination, and the full multi-generational genetic impact of the environment on these populations is unknown.

This study addresses the question of whether an overall increase in DNA mutation rate caused by prolonged, multi-generational exposure to mutagenic compounds is driving the genetic differentiation between these two geographically close populations of free-breeding dogs. In a previous study, we found trends indicative of selective sweeps when comparing the Chornobyl Nuclear Power Plant and Chornobyl City populations [27]. This previous work began to address one of the potential causes for differentiation, selective response, by conducting scans for outlier loci. Here, we investigate an additional possible cause of the genetic differentiation: accumulated germline DNA mutation.

## Materials and methods

### Ethics statement

Ethical review and approval for the animal study was conducted with the permission of the Chornobyl Nuclear Power Plant authorities under the supervision of licensed veterinarians and veterinary technicians. A letter of support detailing the cooperation between the Clean Futures Fund and the Chornobyl NPP was signed by the NPP acting general director V.A. Seyda and can be provided by TAM upon request. Approval for access the exclusion zone was handled and granted by the Exclusion Zone Authority. Data collected for this paper were gathered adventitiously while animals were being treated by the medical program and as such are exempt from IACUC approval.

### Karyotypic architecture

Blood samples from eight dogs, four each from the Nuclear Power Plant and Chornobyl City, were collected into heparin coated tubes during an organized spay, neuter, and vaccination campaign in 2018 conducted by the Clean Futures Fund [27]. Lymphocytes recovered from these blood samples were used to establish short term (72 hour) peripheral lymphocyte cultures, stimulated by a mixture of phytohemagglutinin (PHA) and pokeweed mitogen (PWM), as described previously [29]. Accumulation of cells at metaphase and chromosome preparation were performed [30, 31]. Slides containing chromosome spreads were immersed in 2X saline sodium citrate (SSC) containing 80 ng/mL 4’,6-diaminidino-2-phenylindole (DAPI) for five minutes then washed in 2X SSC before drying and mounting in antifade solution (Vectashield, Vector Laboratories). Metaphase chromosome spreads (at least 30 per dog) were acquired using SmartCapture 3 (Digital Scientific, Cambridge, U.K.), a fluorescence microscope (Olympus BX61) equipped with a Semrock DAPI filter set, and a Hamamatsu ORCA-ER cooled CCD camera (Hamamatsu Photonics). Each DAPI stained metaphase spread was processed in SmartCapture 3.0 using a high-pass spatial filter to reveal enhanced DAPI bands, used for chromosome identification according to the internationally accepted standard karyotype of the domestic dog [30]. The number of chromosomes per nucleus was recorded as well as the sex of each dog according to the visual identification of X and Y chromosomes. Metaphase spreads were exported as TIFF files from SmartCapture 3.0 and imported into SmartType v 3.4.12 (Digital Scientific, Cambridge, U.K) for karyotyping and assessment for the presence of gross translocations or other large-scale mutations.

### Calling and filtering variants

We acquired deidentified raw fastq files from the National Human Genome Research Institute (NHGRI) containing whole genome sequencing (WGS) reads at 20x coverage for 171 dogs residing around the Nuclear Power Plant and in Chornobyl City. We performed a concordance analysis of the WGS data against genotype data from the 714K SNP Axiom Canine HD Array (ThermoFisher; hereafter 714K SNP array) for 116 samples from the Chornobyl Nuclear Power Plant and Chornobyl City populations [27] which determined which samples to retain. The WGS reads were quality trimmed using fastp v 0.21.0 [32] with the default settings and the quality flag -q 20. The trimmed reads were then mapped to the reference genome file GCA_000002285.2_CanFam3.1_genomic.fna (CanFam3 GenBank assembly) using BWA v 0.7.17 [33].

From the resulting alignment files, the properly paired read alignments were saved for downstream use. This was done using samtools v 1.12 [34] and read group information was added using the Picard v 2.25.6 AddOrReplaceReadGroups tool. Using bamtools v 2.5.1 [35], data from different lanes was combined to generate a single bam file for each sample. The bam files were then sorted using samtools before marking duplicates with the Picard MarkDuplicates tool. The GATK v 4.2.0.0 [36] BaseRecalibrator was used to recalibrate the base quality scores for each chromosome, where the known sites came from the 722g.990.SNP.INDEL.chrAll.vcf.1 file that was downloaded from the NCBI Sequence Read Archive (Project: SRP144493).

GatherBQSRReports and ApplyBQSR were used to generate a bam file with the recalibrated data. These recalibrated bam files were retained for identification of variants and also for analysis of short tandem repeat content.

Genomic variants were called using the GATK HaplotypeCaller, which was used to analyze the sequences with the -ERC GVCF flag. The sample output files were combined into one GVCF file per chromosome using CombineGVCFs. The joint genotyping step was run individually for chr1-38 using GenotypeGVCFs. The resulting chromosome VCF files were combined into a single file using bcftools v1.13 [37]. The VCF file was filtered using GATK’s SelectVariants to keep just single nucleotide polymorphisms (SNPs), then filtered using VariantFiltration with the flags: "QD < 2.0", "QUAL < 30.0", "SOR > 3.0", "FS > 60.0", "MQ < 40.0". Only those sites with a PASS flag were saved and vcftools v 0.1.17 [38] was used to select the biallelic SNPs. The final number of SNPs across the 171 samples processed was 13,532,206. The GATK’s SelectVariant tool was used to select only short insertions and deletions (indel) variants, and these were filtered using VariantFiltration with "QD < 2.0", "QUAL < 30.0 ", "FS > 200.0". Only sites with a PASS flag were saved. The SNP VCF was merged with the indel VCF using MergeVcfs.

The 714K SNP array VCF file includes 692,569 autosomal SNPs for 116 samples (detailed in [27]). bcftools isec was used to find SNPs common to both the NHGRI WGS and the 714K SNP array VCF files. This resulted in an intersection of 426,221 SNPs between the two files. To find the number of mismatches between all possible 714K array/NHGRI WGS sample pairings, the bcftools gtcheck command with the flags -e 0—no-HWE-prob was used. The concordance rate between samples was calculated as the proportion of matched SNPs out of the total considered.

Of the 116 samples in the 714k SNP array data set, 106 of the samples had a concordance rate > 96% with at least one sample from the WGS data set. For these 106 samples, 81 of the samples had a single sample match to the WGS set, 23 had two sample matches, and two had three sample matches. KING v 2.3.0 [39] was used to examine the relationship between the SNP array and the WGS samples using the merged 426,221 SNP datasets. This analysis found that the 106 714K SNP array samples with a sample match in the WGS data set all have a relationship that is classified as duplicate/MZ twin. In addition, when more than one WGS sample matched a single 714K SNP array sample, the WGS samples were classified as duplicate/MZ twin. Following the concordance analysis, we retained 106 individuals marked as duplicate/MZ twin.

### Quantification of genetic differentiation

To better assess the degree of genetic differentiation between the Nuclear Power Plant and Chornobyl City populations, we calculated pairwise *F*_*ST*_ between these groups and Eastern European free-breeding dogs. To represent Eastern European free breeding dogs, data comprising CanineHD Whole-Genome BeadChip (Illumina 170k) genotypes of three groups of free breeding dogs originating in Central Russia (N = 16), Eastern Russia (N = 19), and Poland (N = 21) were assembled [40]. These data were combined with a random sample of 20 unrelated individuals from each of the Nuclear Power Plant and Chornobyl City populations to match the sample size of Eastern European dogs. As the Eastern European free-breeding dogs had been genotyped using the CanineHD Whole-Genome Genotyping BeadChip (Illumina), we utilized only those loci overlapping with the Axiom derived genotypes (described in Dillon et al. [27]) present for the Eastern European free-breeding dogs and for the Nuclear Power Plant and Chornobyl City individuals (n = 147,592). We removed all loci with missing genotypes and retained only autosomal variants. We calculated pairwise *F*_*ST*_ between these five populations using Weir and Cockerham’s estimator [41] and established a 99% confidence interval over 1,000 bootstraps through the hierfstat R package [42]. To further investigate these five populations, we conducted a discriminant analysis of principal components (DAPC) using ADEGENET [43]. A K-means procedure was used to identify the optimal number of clusters and assign individuals to these groups. Cross-validation procedures indicated the retention of 20 principal components, and the identified number of clusters was then used to run the analysis and visualize the DAPC results.

### Short tandem repeat (microsatellite) analysis

After assessing genetic differentiation and finding that the Nuclear Power Plant population was equally differentiated from all of the other groups, whereas the Chornobyl City population clustered with the Eastern European dogs, we began to assess mutation using only the Nuclear Power Plant and Chornobyl City population. Should mutation be the driving force for the high levels of genetic differentiation, we would expect to find initial evidence using data from just these two populations. To assess mutation at the genetic level, we considered 54 autosomal STR loci documented to be highly variable (S1 Table); 19 noted as unstable in canine cancers [44, 45], 20 from the International Society for Animal Genetics parentage panels [46], and 15 from DogFiler [47]. STR genotypes for these 54 loci were extracted from recalibrated WGS bam files for all 106 samples from Nuclear Power Plant and Chornobyl City identified through the concordance analysis using GangSTR [48]. GangSTR takes as input a file containing the chromosome, start and end positions, motif length, and the motif and outputs data for each individual at each locus containing genotypes and accompanying likelihood estimations. We retained 94 individuals (Chornobyl City, N = 43; Nuclear Power Plant, N = 51) that group with their respective sample location and form discrete populations (based on cluster analysis in [27]), which allows us to better assess differences between the Nuclear Power Plant and Chornobyl City populations. As a quality filter, we removed 10 STR loci that had low likelihood estimations (L = -25) for more than half of the individuals. We imported the remaining genotypes of 44 high quality loci for 94 individuals into GenAlEx v 6.503 [49, 50] to investigate various genetic diversity measures for the two populations. For each locus, the number of alleles, number of effective alleles, and the number of private alleles were identified, in addition to calculating Shannon’s Information Index, Expected and Observed Heterozygosity, and FIS. GenAlEx was also used to calculate the pairwise *F*_*ST*_ using the STR data. The polymorphic information content (PIC) of each locus was calculated using the PopGenUtils package in R [51]. Additionally, we visualized the allele frequencies of each locus for each population to identify any shifts in allelic frequencies that could be linked to mutational tendencies.

### Count of derived alleles

**Fig 1:**
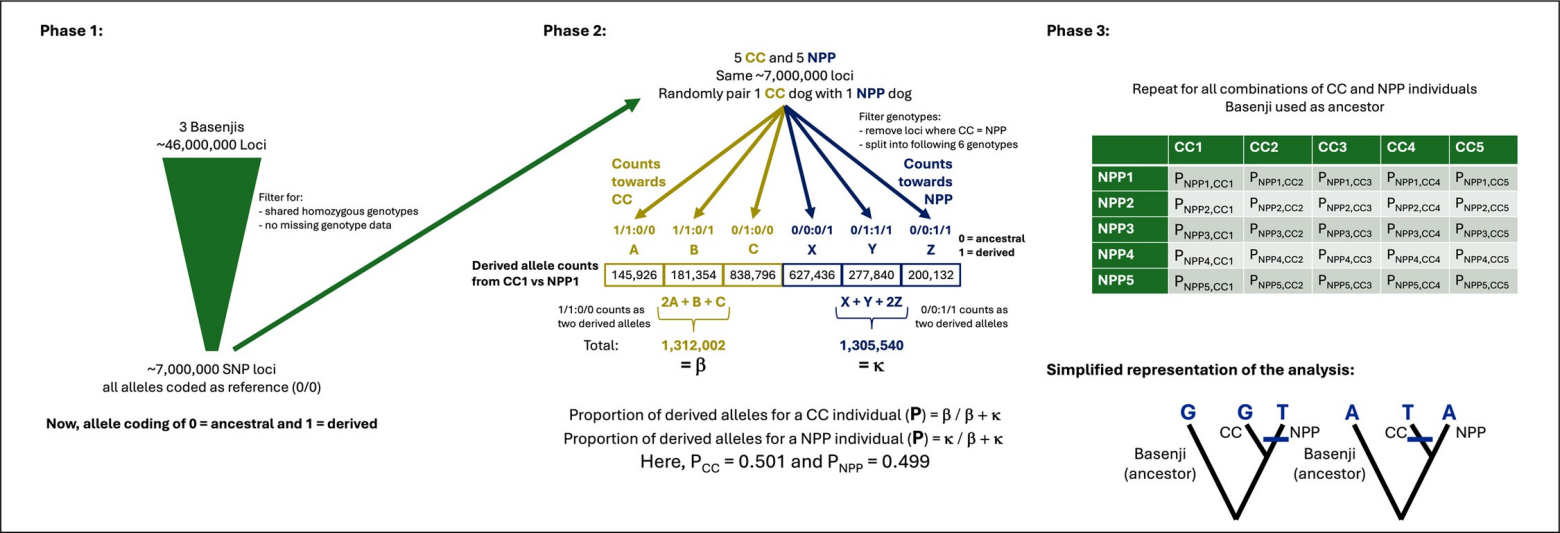
Derived allele analysis pipeline. The analysis involves setting the basenjis as ancestor (Phase 1), counts and proportion calculations for each individual (Phase 2), and summary of the combinatorial pairings for the analysis (Phase 3). CC represents Chornobyl City, NPP represents the Nuclear Power Plant. A simplified representation of derived allele analysis at the single locus level is featured in the lower right, where the ancestor represented here is the basenji. In the derived allele analyses, we scan the genome for instances such as these, where either the PP individual shows evidence of the derived allele (left) or the CC individual contains the derived allele (right) when compared to the ancestor (basenji).

To further investigate mutation, we next examined the WGS data for constitutional base changes at the SNP level. We performed an exploratory analysis that was motivated by the fact that radiation can lead to increased germline mutation rates. Therefore, lineages that are exposed to excess radiation may have more derived alleles than those that are less exposed to radiation. Because basenjis are an ancient breed of dog and one that is not prevalent at the Nuclear Power Plant or Chornobyl City sites based on previous breed analyses [27, 28], we used their genomes to help us categorize which alleles are ancestral and which alleles are derived for this study’s purposes. Specifically, the Chornobyl City and Nuclear Power Plant populations are likely to be more closely related to each other than either is to the basenji breed, allowing the basenji to serve as an outgroup for this analysis. Therefore, when alleles from Chornobyl City and Nuclear Power Plant dogs differ, an allele that is also possessed by the basenjis is likely to be the ancestral state and sharing a genotype is not simply based upon breed-based genetic similarities. Because radiation and other environmental contaminants found within the CEZ could induce new mutation, a finding that one population had a higher proportion of derived alleles than the other would be consistent with more germline change induced by radiation. This analysis followed a pipeline represented in Fig 1 to quantify alleles that had been more recently derived for one group versus the other for each possible pair of Nuclear Power Plant and Chornobyl City dogs.

To begin this analysis, we randomly selected five pairs of unrelated dogs from both the Nuclear Power Plant and Chornobyl City. We then generated a merged VCF file containing genotype data for three basenjis (accessed from [52]) with that of the five randomly selected pairs of Nuclear Power Plant and Chornobyl City individuals using bcftools v 1.13 [37] and removed all loci that had missing data. As genetic variation among basenjis might have existed prior to the splitting of the basenji lineage from the shared ancestral lineage of the Chornobyl City and Nuclear Power Plant dogs, we aimed to lessen the possibility of pre-existing genetic variation persisting up to and after the split between the two populations. To achieve this, we selected only loci for which all three basenjis were homozygous for one allele. PLINK v 2.00a6 [53] was used to recode the three basenji’s major allele (homozygous across the three individual basenjis) for each pair of Nuclear Power Plant and Chornobyl City individuals (—maj-ref,—ref-allele), so as the homozygous basenji alleles were coded as *reference*, and any deviation from the basenjis was coded as *alternate*. For the genomes of each pair of randomly selected Nuclear Power Plant and Chornobyl City dogs, we only considered the polymorphic loci where at least one ancestral (*reference*) allele and at least one derived (*alternate*) allele were present among the four alleles from the two diploid Nuclear Power Plant and Chornobyl city genotypes.

Each pair was assessed for loci with the presence of *reference* (0) and *alternate* (1) alleles in the following orientations– 0/0:0/1, 0/1:1/1, 0/0:1/1, 0/1:0/0, 1/1:0/1, 1/1:0/0 –indicating that one individual in the pair contained a higher number of derived alleles for a locus. For each individual in a pair, we calculated the proportion of derived alleles (denoted as the *alternate* allele). This calculation considered how many more derived alleles the Chornobyl City individual had at a locus when compared to the Nuclear Power Plant individual (1/1:0/0 would count as two derived alleles and both 1/1:0/1 and 0/1:0/0 would count as one derived allele) and vice versa for the Nuclear Power Plant individual. We conducted all possible comparisons for all of the 10 individuals (e.g. Nuclear Power Plant 1 vs Chornobyl City 1, Nuclear Power Plant 1 vs Chornobyl City 2, Nuclear Power Plant 1 vs Chornobyl City 3, etc.) for a total of 25 combinations. To ensure that the five randomly sampled Chornobyl City and Nuclear Power Plant individuals were not misrepresenting the population, we repeated the analysis with additional randomly selected, unrelated Chornobyl City (N = 5) and Nuclear Power Plant (N = 5) individuals. We also repeated this analysis for a new outgroup, the gray wolf (Canis lupus), to ensure that the results were not tied specifically to the basenji serving as the outgroup (accessed from [52]). The analysis was conducted as described previously but included five gray wolves in place of the three basenjis for determination of ancestral vs. derived alleles. We also calculated pairwise *F*_*ST*_ from this dataset containing the three basenjis, five Nuclear Power Plant, and five Chornobyl City individuals to assess the degree of genetic differentiation and ensure that one Chornobyl population was not substantially more differentiated from basenji than the other (PLINK2:—fst method = wc). Pairwise *F*_*ST*_ was also calculated for the five gray wolves, five Nuclear Power Plant, and five Chornobyl City individuals. We are well aware that the relatively small number of generations during which radiation damage from the Chornobyl disaster could accrue means that derived allele proportions are likely to be quite similar in the Chornobyl City and Nuclear Power Plant populations. However, there is a potential of detecting a small but real difference when large genomic data sets are available and considering the high level of genetic differentiation between these populations.

## Results

### Karyotypic architecture

**Fig 2:**
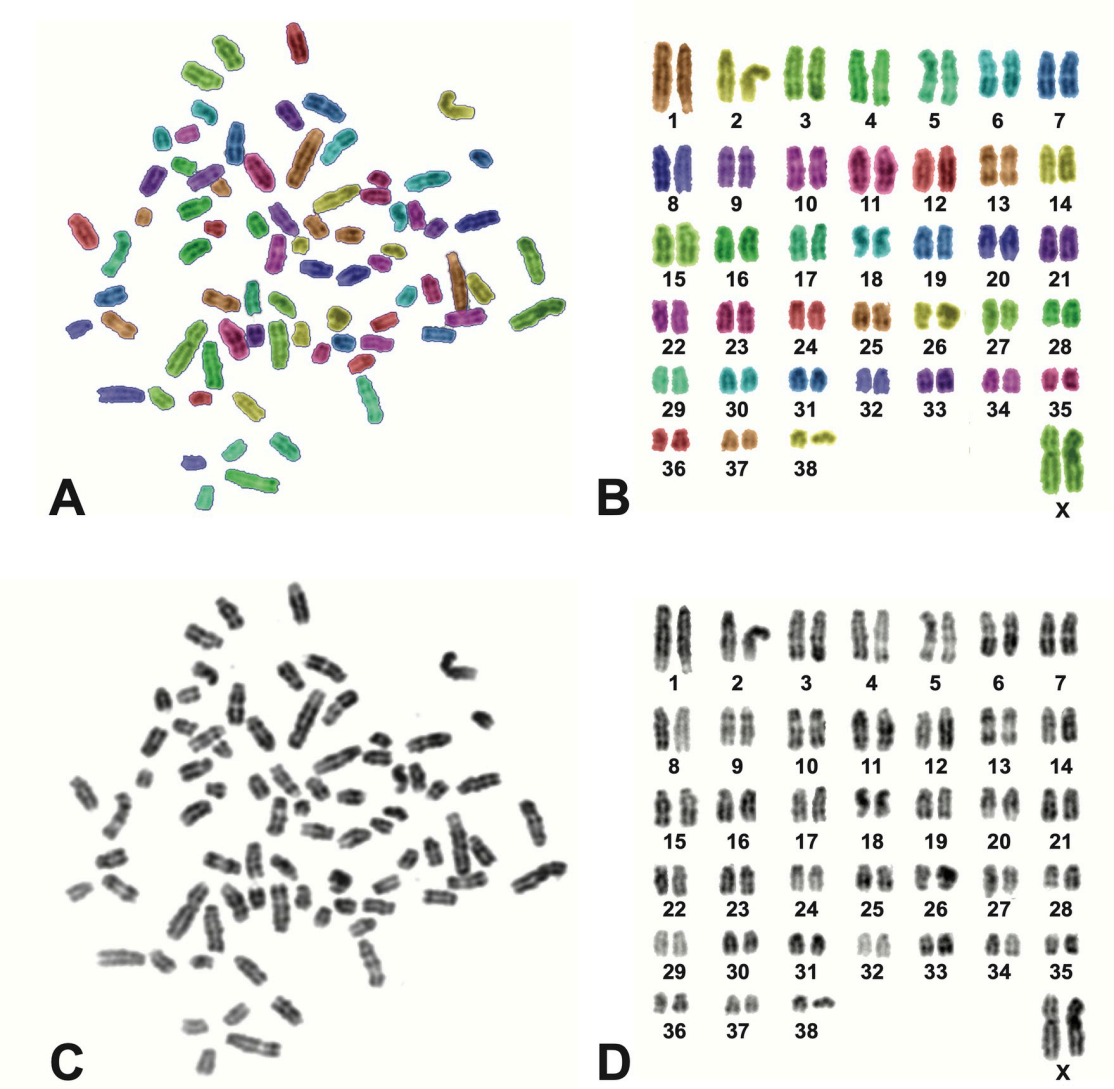
DAPI banded chromosomes of a lymphocyte from a free ranging female dog from the Chornobyl Nuclear Power Plant population. This cell contains 76 autosomes and two X chromosomes, as expected from a domestic dog. (A) and (B) show a DAPI banded metaphase spread and associated karyotype, respectively, tinted to highlight chromosome pairs. (C) and (D) show the DAPI-banded metaphase spread and associated karyotype, respectively, with tinting removed to allow gross cytogenetic evaluation. All cells evaluated had no evidence of gross structural or numerical changes. Tinted metaphases and karyotypes were generated using SmartType v 3.4.12 (Digital Scientific, Cambridge, UK).

As an assessment for larger scale alterations to genomic architecture, we analyzed metaphase spreads from a subset of dogs in the study. Of the eight blood samples obtained for peripheral lymphocyte culture, seven (four Nuclear Power Plant and three Chornobyl City individuals) produced sufficient quality metaphase spreads to permit chromosome enumeration from at least 30 nuclei. In all seven dogs, chromosome counts for contained, discrete metaphases yielded 76 autosomes and two sex chromosomes per individual cell, across at least 30 metaphases per dog. The chromosomal sex of each of the seven dogs was fully concordant with the sex by genotyping and metadata for each. We did not note any gross chromosomal abnormalities in any of the seven dogs assessed during a visual sweep of their karyotypes. A representative karyotype of one dog, a female from the Nuclear Power Plant, is shown in Fig 2.

### Calling and filtering variants

A total of 106 individuals from the WGS data set had concordance with the 714K SNP array genotypes from our Axiom data set and were retained. Following the filtering of SNPs and indels for these 106 individuals, the resultant file contained 18,455,110 total variants across the genome, including 13,532,206 SNPs and 4,922,904 indels.

### Quantification of genetic differentiation

**Fig 3:**
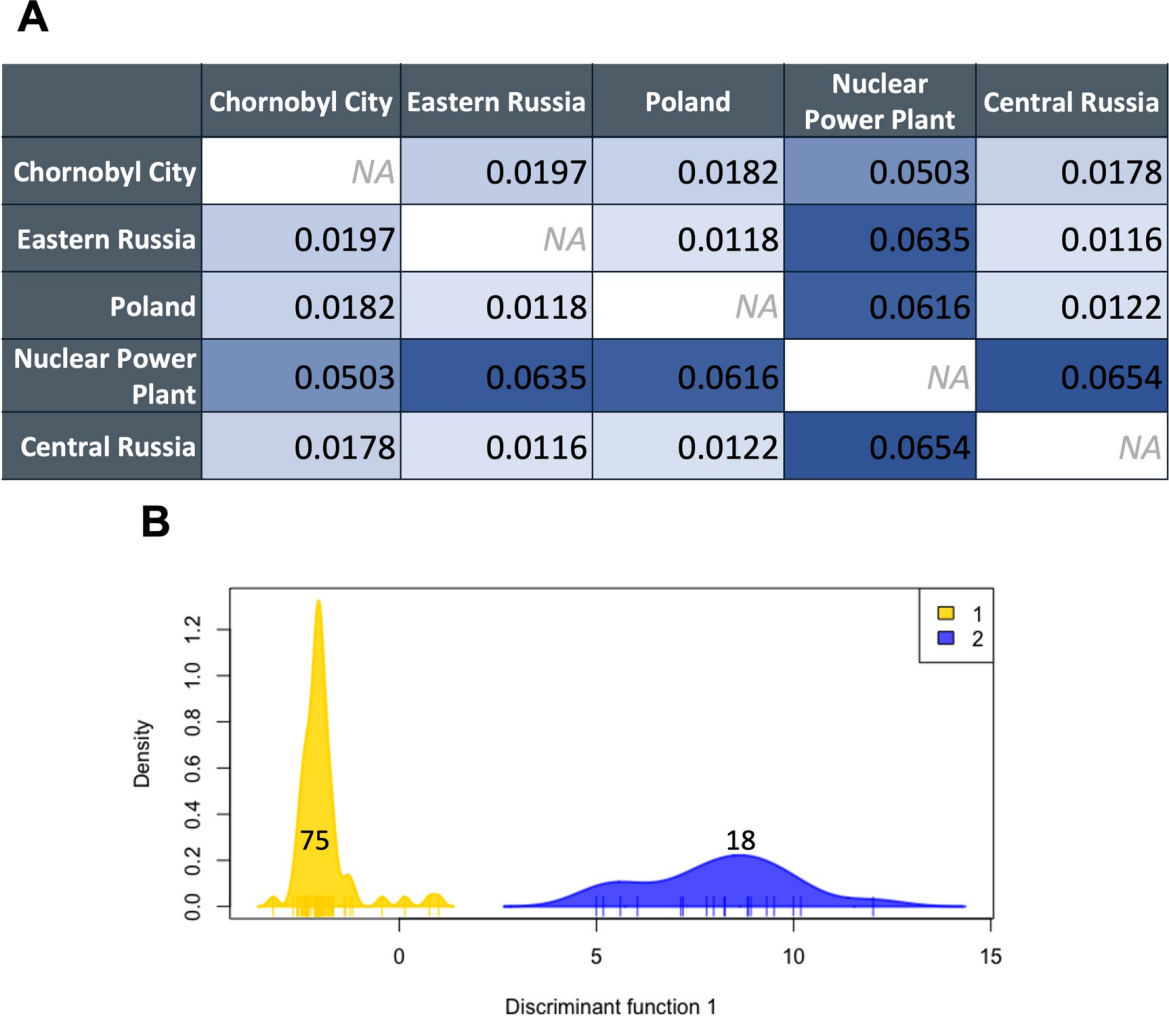
Genetic differentiation analyses for Chornobyl and Eastern European free-breeding dog populations. (A) Pairwise *F*_*ST*_ measures between all five populations, color scales from low *F*_*ST*_ (white) to high *F*_*ST*_ (blue). All *F*_*ST*_ values are significant based on bootstrapping to 99% confidence interval. (B) Cluster analysis and subsequent discriminant analysis of principal components, where cluster two (blue) is entirely Nuclear Power Plant individuals and cluster one (gold) contains the other four groups.

When we assessed genetic differentiation with SNP data and calculated pairwise *F*_*ST*_ for equal numbers of unrelated dogs residing at the Nuclear Power Plant, Chornobyl City, Eastern Russia, Central Russia, and Poland, we found comparable levels of differentiation between the Nuclear Power Plant population and all other groups (Fig 3A). All values of *F*_*ST*_ were significant, as the 99% confidence intervals failed to include zero. The Chornobyl City population, however, is less differentiated from the Eastern European groups outside of Chornobyl. This finding is echoed in the results of the Discriminant Analysis of Principal Components (DAPC), where we note two distinct clusters: one made up entirely of Nuclear Power Plant individuals and the other made up of the four other free-breeding dog populations (Fig 3B). Despite the geographic distance between the other four groups, they share more genetic similarity to each other than to the Nuclear Power Plant population. A slight degree of overlap lies between the Nuclear Power Plant and Chornobyl City populations, as two Nuclear Power Plant individuals cluster with the opposite group. The analysis resulted in one cluster with 18 individuals (all Nuclear Power Plant) and the other cluster containing the 75 remaining individuals (two Nuclear Power Plant, 20 Chornobyl City, 16 Central Russia, 19 Eastern Russia, and 21 Poland).

### Short tandem repeat (microsatellite) analysis

**Fig 4:**
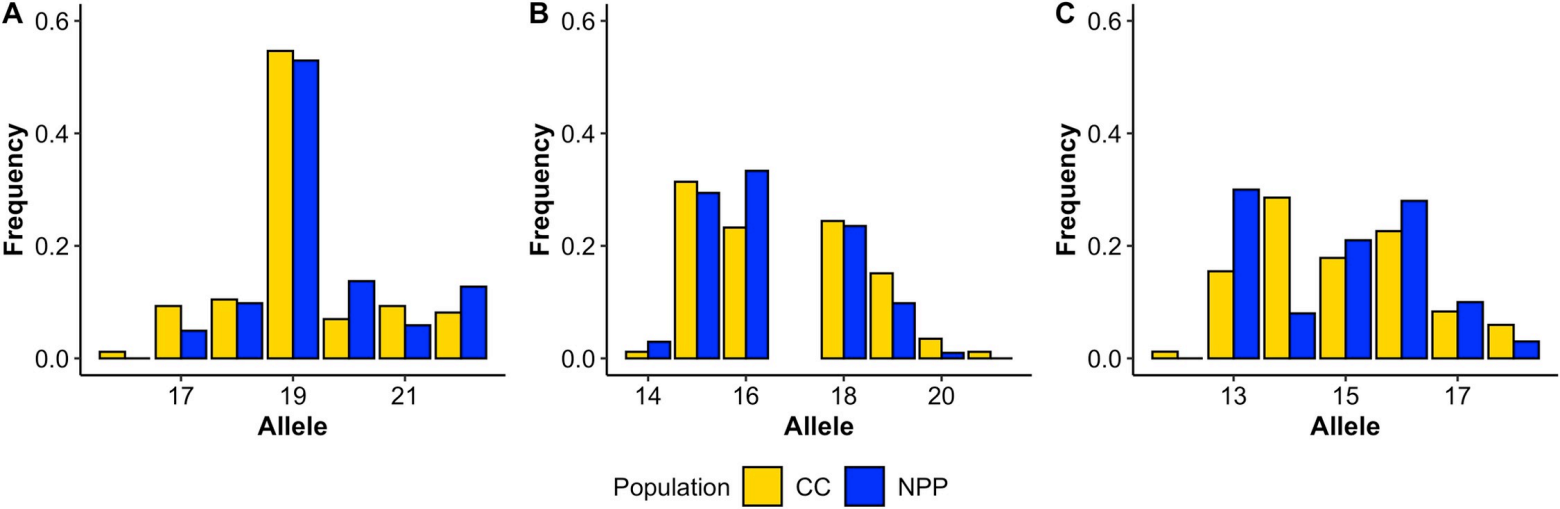
Representative selection of allele frequency distributions for three loci. Included here are (A) AHTk253, (B) CDH4, (C) VGL2409. Chornobyl City frequencies indicated in gold, Nuclear Power Plant in blue. More plots are available in S1 Fig.

After noting the high level of genetic divergence between the Nuclear Power Plant population and all other considered populations, we considered only the Nuclear Power Plant and Chornobyl City group for the following analyses. We first looked at 44 short tandem repeat (STR) loci for evidence of an elevated mutation rate for the Nuclear Power Plant population. With this analysis, we looked for evidence of accumulated mutation by investigating different genetic diversity estimates for the two considered populations. In investigating the STRs, a similar level of genetic differentiation was noted between these two populations for STR genotypes and for that of the SNP genotypes (STR *F*_*ST*_ = 0.047; SNP *F*_*ST*_ = 0.050). All 44 loci considered were polymorphic, with an average of 6.7 alleles per locus overall. A significantly higher average number of alleles, effective number of alleles, and number of private alleles are found within the Chornobyl City population than in the Nuclear Power Plant population, as well as a significantly higher level of expected heterozygosity for the Chornobyl City population (Table 1, p < 0.05 by paired t-test). The observed heterozygosity and inbreeding coefficient (*F*_*IS*_), however, are higher within the Chornobyl City population but the differences are not significant. Shannon’s information index and the polymorphic information content are both significantly higher for the Chornobyl City population. We do not discern any notable trends in allele frequency distributions at each locus, including shifts towards larger or smaller alleles (e.g., Figs 4 and S1).

**Table 1:** STR diversity measures for the Nuclear Power Plant and Chornobyl City populations.

Population		No. Individuals	No. Alleles	Effective No. Alleles	Shannon’s Information Index	H_O_	H_E_	F_IS_	No. Private Alleles	PIC
**Chornobyl City**	**Mean**	42.422	7.356*	4.284*	1.561*	0.621	0.718*	0.136	1.733*	0.685*
	**SE**	0.175	0.320	0.255	0.060	0.026	0.021	0.025	0.166	0.022
**Nuclear Power Plant**	**Mean**	49.822	6.133	3.333	1.339	0.583	0.660	0.114	0.511	0.618
	**SE**	0.329	0.255	0.164	0.050	0.023	0.020	0.024	0.108	0.021
**Overall**	**Mean**	46.122	6.744	3.809	1.450	0.602	0.689	0.125	—	0.675
	**SE**	0.434	0.214	0.159	0.041	0.017	0.015	0.017	—	0.0212

Ho = observed heterozygosity, He = expected heterozygosity, F_IS_ = fixation index, PIC = Polymorphic Information Content. Asterisk (*) indicates difference between the two populations generates a p < 0.05 by t-test.

### Count of derived alleles

**Fig 5:**
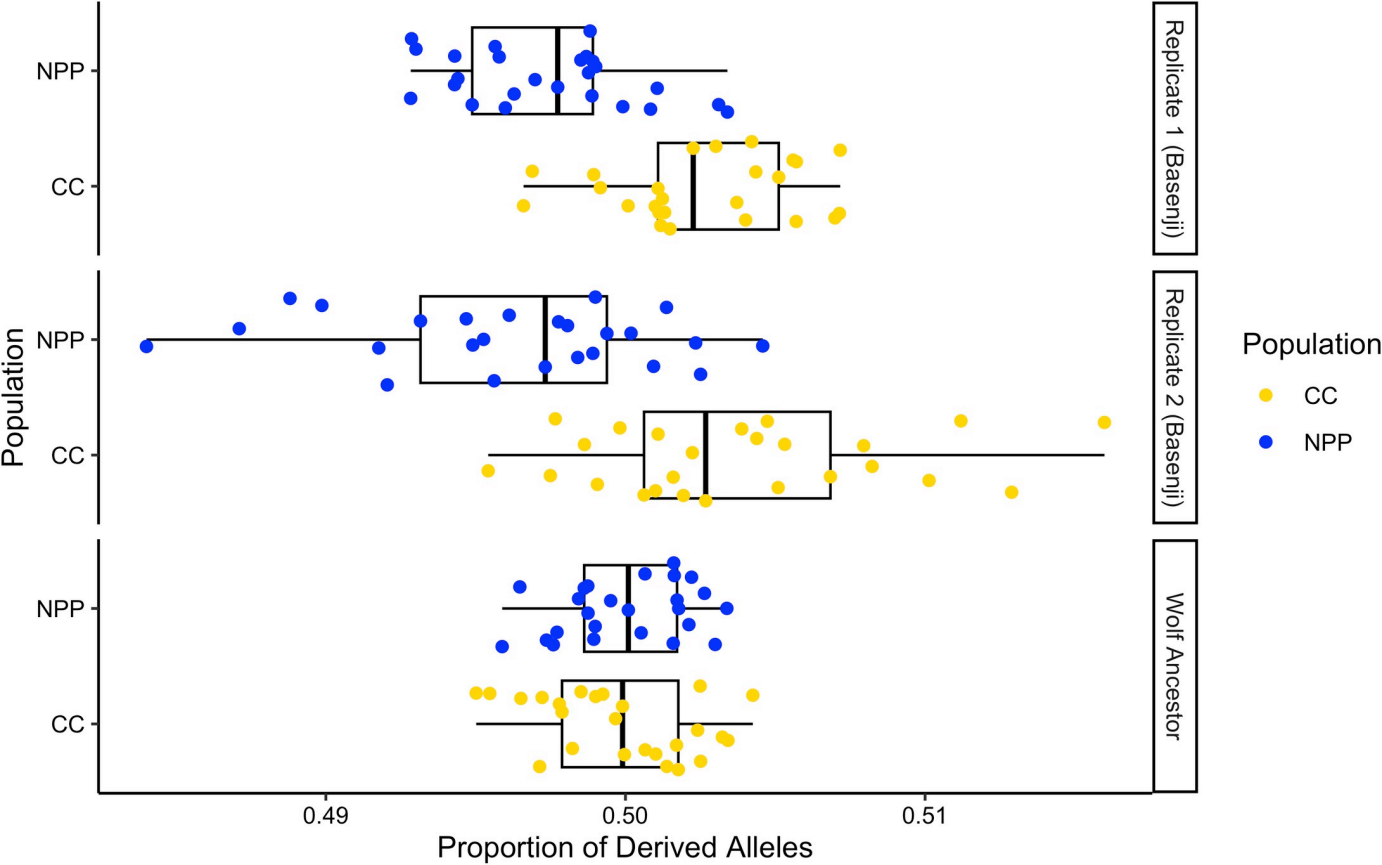
Summary of derived allele proportions for all 25 possible pairs of Nuclear Power Plant (NPP) and Chornobyl City (CC) individuals for all three analyses. These plots depict Replicate one and Replicate two, with basenji as the common ancestor, and the final trial with gray wolf serving as the ancestor. Box plots highlight lower quartile, median, and upper quartile, and the whiskers extend to the most extreme values but not further than 1.5 * inter-quartile range (IQR) from the hinge. Points indicate the true proportions taken for each comparison.

With the derived allele analysis, we assessed the WGS data for a higher-than-expected proportion of derived alleles in one population over the other, which could be indicative of an increased mutation rate. For 20 of the 25 combinations of Nuclear Power Plant and Chornobyl City individuals, the Chornobyl City had a slightly higher proportion of derived alleles, despite varying across the pairs (Fig 5 and S2 Table). Overall, the proportion of derived alleles across the Chornobyl City population was higher than that of the Nuclear Power Plant when using the basenji as the ancestor in both replicates (Mean proportion of derived alleles; Replicate 1: Chornobyl City = 0.503, Nuclear Power Plant = 0.497, Std. dev. = 0.003; Replicate 2: Chornobyl City = 0.504, Nuclear Power Plant = 0.496, Std. dev. = 0.005), and the proportion was not different between populations when using the gray wolf as ancestor (Chornobyl City = 0.500, Nuclear Power Plant = 0.500, Std. dev. = 0.003). Calculating pairwise *F*_*ST*_ for the subsampled groups highlighted similar levels of genetic differentiation between the basenjis against the Nuclear Power Plant population and against the Chornobyl City population (Nuclear Power Plant vs Basenji = 0.339, Chornobyl City vs Basenji = 0.329), and also between these two Chornobyl populations and the wolves (Nuclear Power Plant vs Gray Wolves = 0.223, Chornobyl City vs Gray Wolves = 0.208).

## Discussion

The 1986 disaster at the Chornobyl Nuclear Power Plant resulted in contamination of the surrounding environment with a wide variety of mutagens, and we have a poor understanding of how this affected mutation in local communities. In this study, we assessed mutation rate and accumulated mutations in two established populations of free-breeding dogs that live within the CEZ. Two previous studies reported significant genetic differentiation between these two geographically close free-breeding dog populations at the Chornobyl Nuclear Power Plant and Chornobyl City, as well as differentiation between the Nuclear Power Plant population and dog populations outside of the CEZ [27, 28]. Previous assessments of acquired mutations linked to the contamination of this disaster for Chornobyl based populations have yielded conflicting results across different species. Tintori et al. [54] found no dose-related correlation between that the number of private mutations, radioisotope levels, or distance to the Nuclear Power Plant, in a worm species native to the CEZ. A comprehensive and detailed examination of germline de novo mutation in children of Chornobyl cleanup workers, which considered the entire trio of father, mother, and offspring, found no association between mutation and parental radiation exposure [55]. In contrast, Goodman et al. [56] and Baker et al. [57], reported increased genetic diversity associated with radiation exposure in populations of Daphnia and populations of bank voles, respectively. Given the diversity of and contrast between these findings, it is important to examine mutation rates in other exposed animal populations within the CEZ. It is notable, therefore, that the present study did not find evidence of an increased mutation rate or accumulated mutations in either of the two modern, free-breeding dog populations living in the Chornobyl Exclusion Zone.

Our study provides additional support for the previous finding that the free-breeding dogs located at the Nuclear Power Plant are genetically differentiated from the population only 16 km away in Chornobyl city, and that the Nuclear Power Plant population is differentiated from other dog populations outside the CEZ. Pairwise *F*_*ST*_ analysis and the DAPC highlight genetic differentiation between the Nuclear Power Plant population and populations of free-breeding dogs in Chornobyl City, Eastern Russia, Central Russia, and Poland. It is interesting to note that the dogs assessed from Chornobyl City, however, are more genetically similar to those representing Russian and Polish populations. Comparing the Nuclear Power Plant and Chornobyl City populations with DNA sequence-based mutation analyses, therefore, allowed us to assess whether mutation is driving the differentiation between the Nuclear Power Plant population and other free-breeding dog populations. With the inclusion of additional groups for comparison and increased power with our expanded data set, we extend previous findings on the distinctiveness specifically of the Nuclear Power Plant population [27, 28].

Despite confirming that these free-breeding dogs at the Nuclear Power Plant are genetically differentiated from other groups, we did not find evidence of an increased DNA mutation rate or increased mutation accumulation between the Nuclear Power Plant and Chornobyl City populations. Unlike the findings for the Chornobyl Daphnia populations [56], we do not find an increase in private alleles, effective alleles, or heterozygosity for STR/microsatellite loci within the Nuclear Power Plant dog population. The Nuclear Power Plant does not show evidence of increased genetic diversity compared to the Chornobyl City population, and increased genetic diversity would be expected to accompany an increased rate of mutation accumulation. We also do not find evidence for an increased proportion of derived alleles for the Nuclear Power Plant population when comparing this population to the Chornobyl City population. Because we assume that the founder populations of the Nuclear Power Plant and Chornobyl City dogs to be similarly related to the basenji, we would expect a proportion of derived alleles of 0.50 for each population under neutrality. We do not find an increase in this proportion for the Nuclear Power Plant population, but rather find that the Chornobyl City population has a slightly higher proportion of derived alleles on average across the different analyses. These populations do not have significantly different levels of inbreeding based on our analyses, therefore these trends in both STR/microsatellite diversity and derived alleles are not seemingly tied to a higher level of inbreeding in one population. These data do not indicate that the Nuclear Power Plant has a higher proportion of recently accumulated variants, and therefore do not indicate a higher mutation rate.

In addition to the lack of evidence towards mutation accumulation changes at a fine scale we looked for any gross changes to karyotypic architecture within a subset of dogs from the Nuclear Power Plant and Chornobyl City. Although this was a small sample size (four dogs at the Nuclear Power Plant and three at Chornobyl City), we did not observe any gross chromosomal abnormalities. Overall, through these analyses, we do not find evidence for higher mutation accumulation or mutation rate at the distinct Nuclear Power Plant population, despite the higher level of environmental contamination found there.

The conclusions drawn from the free-breeding dogs around the Chornobyl Nuclear Power Plants corroborates trends identified through recent work on local worm species and investigations of the children of Chornobyl cleanup workers [54, 55]. While we were limited in our ability to access dog samples from before the accident to allow for the trio assessments utilized in Yeager et al. [55], our analyses consider both large- and small-scale evidence for assessing genome-wide mutation. Our findings for these Nuclear Power Plant dogs are inconsistent with an increased mutation rate or high levels of mutation accumulation, despite the higher levels of contamination surrounding the power plant itself [17, 58]. Thus, an elevated mutation rate does not appear to be the cause of the genetic divergence of the Nuclear Power Plant population highlighted in Dillon et al. [27].

## Conclusions

With this study, we do not find evidence of an increased mutation rate for the Nuclear Power Plant population of dogs through chromosomal aberrations, increased microsatellite diversity, or an increase of more recently derived alleles. Therefore, mutation does not appear to be the cause of the previously identified genetic differentiation between these two geographically close populations of free-breeding dogs. Considering this, in conjunction with the previous work on breed composition, inbreeding, and comparisons to other free-breeding dog populations, we have yet to identify the definitive cause for this genetic differentiation [27]. However, we did previously identify genomic regions with allele frequencies suggestive of directional selection between the Nuclear Power Plant and the Chornobyl City populations, including candidate loci in close proximity to genes involved in regulation of the cell cycle and response to DNA damage. With these new findings, there is support for the hypothesis that selection may be helping to drive the genetic divergence of these populations. Therefore, additional investigation is required to address this in addition to assessing whether differences are due to environmental exposure factors, considering the contrast in levels of contamination between the Nuclear Power Plant and Chornobyl City sample sites. Findings from such studies are likely to inform other studies of multi-generational exposure to various environmental contaminants in other animal species, flora, and humans working within the CEZ, and can also be applied to other communities in areas of environmental contamination.

## Supporting information

S1 Fig Allele frequency distributions for all 44 considered STR loci. The size of the allele is on the x-axis, and frequency per population on the y-axis. Blue denotes frequencies for the Nuclear Power Plant (NPP) population, and gold indicates frequencies for Chornobyl City (CC).
(DOCX)

S1 Table Microsatellite/STR loci included in diversity analysis. Included are the 44 loci used for analysis and represented in order in S1 Fig.
(XLSX)

S2 Table Derived allele counts and proportions for all pairs of Nuclear Power Plant (NPP) and Chornobyl City (CC) for each analysis. Each table includes the genotype counts and the count of derived alleles for each individual in a pair, along with each calculated proportion. Replicate 1 and 2 both use the basenji as the ancestor, while the third analysis uses the wolf. Pair indicates the original, randomly designated pairings, while mixed pair indicates the subsequent possible pairings.
(XLSX)
