# Supplementary material for: Is increased mutation driving genetic diversity in dogs within the Chornobyl exclusion zone?
Source: PLoS One. 2024 Dec 27;19(12):e0315244. doi: 10.1371/journal.pone.0315244 (PMC11676578; doi:10.1371/journal.pone.0315244)
Supplement: S1 Fig — The size of the allele is on the x-axis, and frequency per population on the y-axis. Blue denotes frequencies for the Nuclear Power Plant (NPP) population, and gold indicates frequencies for Chornobyl City (CC). (DOCX) [file pone.0315244.s001.docx]

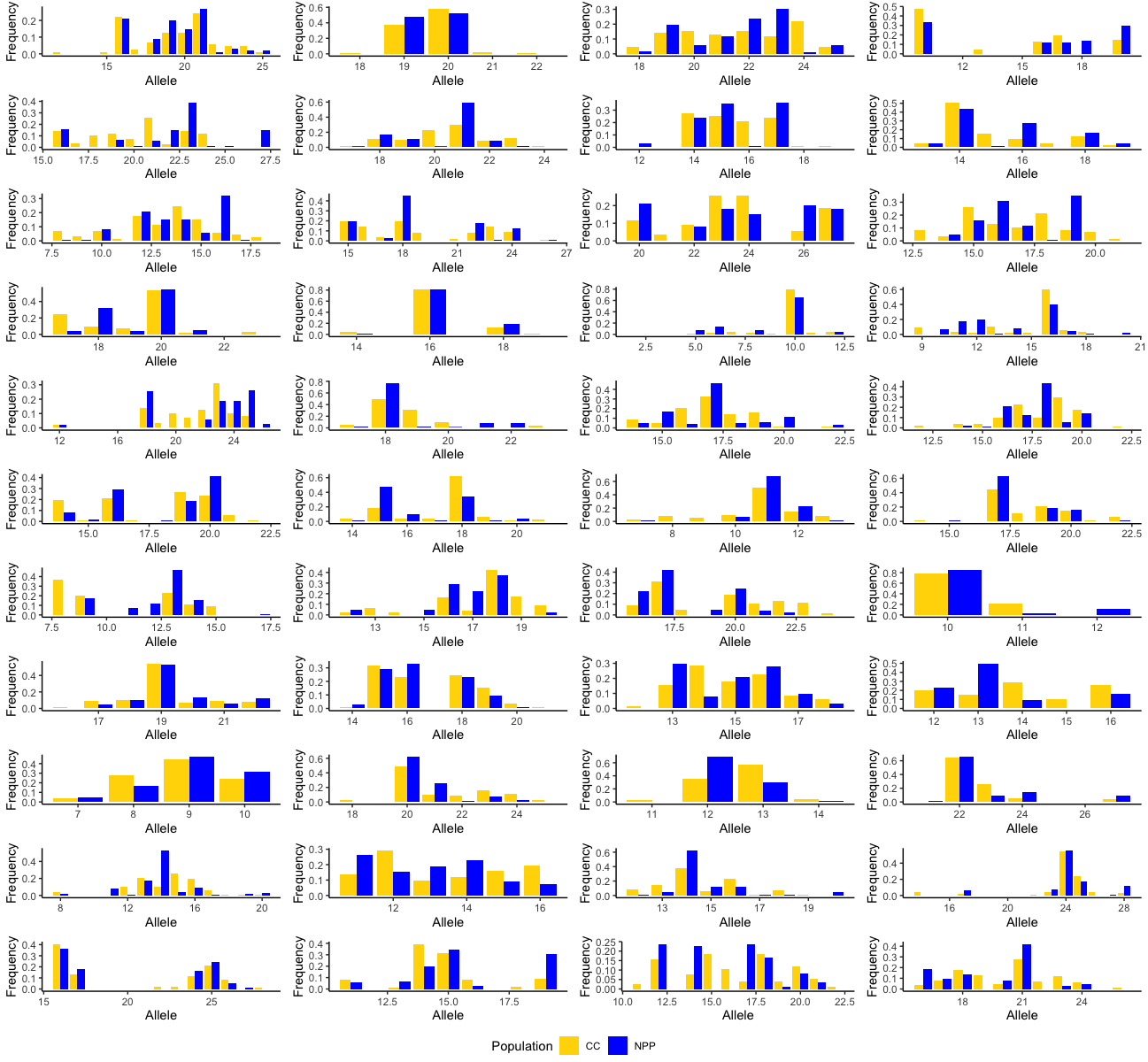


Fig. S1. Allele frequency distributions for all 44 considered STR loci. The size of the allele is on the x-axis, and frequency per population on the y-axis. Blue denotes frequencies for the Nuclear Power Plant (NPP) population, and gold indicates frequencies for Chornobyl City (CC).
